# Gallic Acid Attenuates Platelet Activation and Platelet-Leukocyte Aggregation: Involving Pathways of Akt and GSK3**β**

**DOI:** 10.1155/2012/683872

**Published:** 2012-06-28

**Authors:** Shih-Sheng Chang, Viola S. Y. Lee, Yu-Lun Tseng, Kuan-Cheng Chang, Kuen-Bao Chen, Yuh-Lien Chen, Chi-Yuan Li

**Affiliations:** ^1^Graduate Institute of Clinical Medical Science, China Medical University, Taichung 40402, Taiwan; ^2^Division of Cardiology, Department of Medicine, China Medical University Hospital, Taichung 40407, Taiwan; ^3^Department of Psychiatry, China Medical University Hospital, Taichung 40407, Taiwan; ^4^Department of Anesthesiology, China Medical University Hospital, Taichung 40407, Taiwan; ^5^Graduate Institute of Anatomy and Cell Biology, College of Medicine, National Taiwan University, Taipei 10051, Taiwan

## Abstract

Platelet activation and its interaction with leukocytes play an important role in atherothrombosis. Cardiovascular diseases resulted from atherothrombosis remain the major causes of death worldwide. Gallic acid, a major constituent of red wine and tea, has been believed to have properties of cardiovascular protection, which is likely to be related to its antioxidant effects. Nonetheless, there were few and inconsistent data regarding the effects of gallic acid on platelet function. Therefore, we designed this *in vitro* study to determine whether gallic acid could inhibit platelet activation and the possible mechanisms. From our results, gallic acid could concentration-dependently inhibit platelet aggregation, P-selectin expression, and platelet-leukocyte aggregation. Gallic acid prevented the elevation of intracellular calcium and attenuated phosphorylation of PKC**α**/p38 MAPK and Akt/GSK3**β** on platelets stimulated by the stimulants ADP or U46619. This is the first mechanistic explanation for the inhibitory effects on platelets from gallic acid.

## 1. Introduction

Platelets are essential for primary hemostasis and the repair of endothelium, but they also play a key role in the development of acute coronary syndromes and contribute to cerebrovascular events. Platelet activation triggered by inflammation is the critical component of atherothrombosis [1]. In addition, platelets participate in the process of forming and extending atherosclerotic plaques [2]. When activated, platelets coaggregate with circulating leukocytes via P-selectin glycoprotein ligand-1 (PSGL-1) and P-selectin interactions. These interactions trigger autocrine and paracrine activation processes leading to the recruitment of the leukocytes into the vascular wall, which is important in the formation of atherothrombosis [3]. In a large-scale prospective human study, the risk of future cardiovascular events increased with increasing levels of plasma platelet-leukocyte aggregation [4]. 

Gallic acid (3,4,5-trihydroxybenzoic acid), a naturally occurring plant phenol, which can be abundantly found in natural plants, tea, and red wines [5], has been demonstrated to have various biological properties, including antioxidant [6], anticancer [7], and anti-inflammatory activities [8]. Epidemiological studies have suggested that red wine consumption is related to a reduction in overall mortality [9]. Although the exact nature of the protective effect of red wine is unclear, it might be partially attributed to its ability to reduce the progression of atherosclerotic lesions [10]. Green tea has also been reported to have protective effects on cardiovascular diseases [11]. Gallic acid itself has been shown to protect the myocardium against isoproterenol-induced oxidative stress in rats [12]. Previous reports on the favorable effects of gallic acid focused on its anti-oxidant and anti-inflammatory properties [8, 13], but it remains unknown whether or not gallic acid is atheroprotective through nonantioxidant mechanisms, for example, through inhibiting platelet activation. Up to date, there have been only scanty and inconsistent data concerning the effects of gallic acid on platelet function. Therefore, the purpose of our study was to determine whether gallic acid could inhibit platelet function *in vitro* and to elucidate the underlying molecular mechanisms. 

## 2. Materials and Methods

### 2.1. Antibodies and Reagents

The following antibodies were used: anti-CD42a-PE antibody (Becton Dickinson, San Jose, CA, USA), a platelet-specific monoclonal antibody (mAb) conjugated with phycoerythrin (PE), which recognizes platelet glycoprotein IX complex independent of activation, anti-CD62P-PE antibody (Becton Dickinson), an mAb conjugated with PE that is directed against P-selectin expressed on the platelet surface, and anti-CD14-allophycocyanin (APC) antibody (Becton Dickinson), an mAb which recognizes a myelomonocytic differentiation antigen expressed by monocytes. Polyclonal antibodies against p38 mitogen-activated protein kinase (MAPK), protein kinase C-alpha (PKC*α*), and Akt were obtained from Cell Signaling (Boston, MA, USA). Polyclonal antibodies against glycogen synthase kinase-3*β* (GSK3*β*) were purchased from R&D Systems (Minneapolis, MN, USA). 2′7′-dichlorodihydrofluorescein diacetate (DCFH-DA) and fluo-3 acetoxymethyl ester (fluo-3 AM) were obtained from Molecular Probes (Eugene, OR). Adenosine 5′-diphosphate (ADP), gallic acid, and paraformaldehyde were purchased from Sigma Chemicals (St. Louis, MO, USA). U46619, a thromboxane A_2_ (TxA_2_) mimetic, was obtained from Cayman Chemical (Ann, Arbor, Michigan, USA). Gallic acid was dissolved in dimethylsulfoxide (DMSO). Steps were taken to ensure that the concentration of DMSO remained the same (0.1%). 

### 2.2. Preparation of Platelet Suspension

Human platelets were purified as previously described [14]. Whole blood for the *in vitro* study was sampled from six healthy volunteers with age ranging from 27 to 53 years, who had not taken any medication for at least 15 days. Blood was collected from the antecubital vein into acid-citrate-dextrose (9 : 1) and centrifuged at 200 ×g for 20 minutes at 25°C to prepare platelet-rich plasma (PRP). PRP was first washed with modified Tyrode's solution (NaH_2_PO_4_: 0.42 mM, NaCl: 136.9 mM, KCl: 2.68 mM; NaHCO_3_: 11.9 mM; CaCl_2_: 1.85 mM; MgCl_2_: 1.0 mM; 0.35% BSA and 0.1% glucose) containing heparin (7 U/ml) and PGE_1_ (0.6 *μ*M) and centrifuged at 600 ×g for 15 minutes at 25°C. After descanting the supernatant, pellet was then washed twice with modified Tyrode's solution containing heparin and PGE_1_. Finally, washed platelets were resuspended to a final concentration of 3 × 10^8^ platelets/ml in Tyrode's solution containing 0.35% BSA and incubated at 37°C.

### 2.3. Platelet Aggregation

Platelet aggregation was measured with an aggregometer (Payton Scientific, Buffalo, NY, USA) as previously described [15]. Briefly, PRP was applied to the aggregometer and stirring was initiated at 900 rpm for 1 minute at 37°C with a small magnetic bar. Then, various concentrations of indicated gallic acid were added and incubated for 3 minutes followed by adding proaggregatory substance ADP (2.5 *μ*M) and TxA_2_ analog U46619 (1.5 *μ*M). We used PowerLab 8/SP (ADInsturments, Sydney, Australia) to analyze the extent of platelet aggregation that was continuously monitored for 8 minutes by turbidimetry and expressed as increase of light transmission.

### 2.4. Assess Platelet-Leukocyte Aggregates and P-Selectin Expression by Flow Cytometry

The amount of platelet-leukocyte aggregates (PLAs) and P-selectin expression on platelets was determined by cytofluorimetric analysis. Anticoagulated whole blood and PRP were preincubated with the indicated concentration of gallic acid for 15 minutes at 37°C. The blood samples were treated for 15 minutes of stimulation at room temperature with ADP and U46619 at a concentration of 2 *μ*M in whole blood and 5 *μ*M in PRP. To determine PLA, whole blood was mixed with saturated concentrations of anti-CD42a-PE mAb and anti-CD14-APC mAb. To determine platelet P-selectin expression, PRP samples were mixed with saturated concentrations of anti-CD62p-PE mAb and anti-CD42a-PE mAb. Both samples were then fixed with 1% paraformaldehyde and maintained at 4°C. After fixation, blood samples were immediately processed for flow cytometric analysis in a FACSCanto (Becton Dickinson). Granulocytes were recognized by size (forward scatter) and granularity (side scatter). Anti-CD14-APC fluorescence was used to further differentiate monocytes. The amount of platelets attached to granulocytes and monocytes was further measured by the anti-CD42a fluorescence. To determine platelet CD62P expression in PRP, individual platelets were identified by size (forward scatter) and anti-CD42a-PE immunofluorescence. P-selectin expression on the surface of platelets was defined as positive for anti-CD62P-PE. Results are expressed as mean fluorescence intensity (MFI) and percentage of positive CD62P cells.

### 2.5. Measurements of Intracellular Ca^**2**+^ Concentration

Intracellular Ca^2+^ levels were determined with the Ca^2+^-sensitive fluorochrome fluo-3 AM using flow cytometry as previously described [16]. Briefly, washed human platelets (3 × 10^8^ platelets/mL) were loaded with 8 *μ*M fluo-3 AM for 30 minutes at 37°C in the dark. After being washed once, platelets were resuspended and the external Ca^2+^ was adjusted to 1 mM and then the dyed platelets were incubated with ADP (10 *μ*M) or U46619 (2 *μ*M) and different concentrations of gallic acid (100 *μ*M, 500 *μ*M) or control vehicles at 37°C for 3minutes in the dark and analyzed by flow cytometry. 

### 2.6. Determination of Reactive Oxygen Species Formation

The influence of gallic acid on reactive oxygen species (ROS) production of stimulated platelets was tested by 2′7′-dichlorodihydrofluorescein diacetate (DCFH-DA) (Molecular Probes, Eugene, OR) and flow cytometry as previously described [17]. In brief, PRP was preloaded with 10 *μ*M DCFH-DA for 30 minutes at 37°C followed by stimulation of U46619 (2 *μ*M). Oxidation was quantified by measuring the increase in fluorescence of 2′7′-dichlorodihydrofluorescein (DCF) with a flow cytometer. The effect of gallic acid on ROS production of platelets was decided by pretreatment of gallic acid of the indicated concentration for 15 minutes before adding U46619. 

### 2.7. Western Blot Analysis

The method of western blotting was performed as previously described [18]. Various concentrations of indicated gallic acid were added to PRP and incubated for 3 minutes followed by adding proaggregatory substance ADP (2.5 *μ*M). The reaction was stopped with 2 *μ*M EDTA and 50 *μ*M Indomethacin. Proteins were extracted with lysis buffer for 30 minutes. Lysates were centrifuged, and the supernatant (46.4 *μ*g protein) was subjected to SDS PAGE (10%) and blotted on PVDF membrane. Immunodetection was carried out by using antibodies directed against phosphorylated and origin forms of p38 MAPK, PKC*α*, Akt, and GSK3*β*. The immunoreactive band was detected by enhanced chemiluminescence.

### 2.8. Statistical Analysis

All data were expressed as the mean ± standard error of the mean. Each sample was compared with the corresponding control sample. Analysis of statistical significance was performed using one-way analysis of variance (ANOVA) combined with the Turkey test. For comparison between two groups Student's *t* test was used. *P* < 0.05 was considered to be significant for a difference.

## 3. Results

### 3.1. Gallic Acid Inhibits Platelet Aggregation

**Figure 1:**
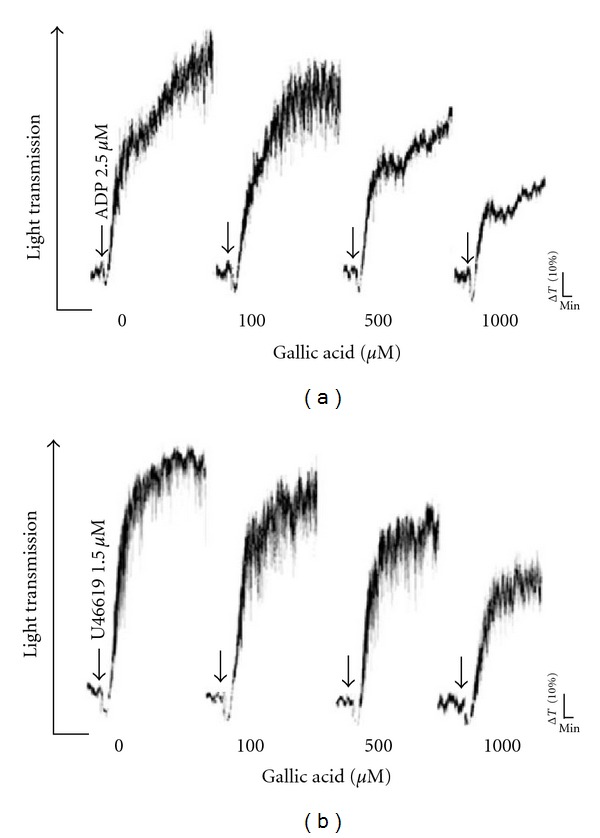
Inhibitory effects of gallic acid on ADP- or U46619-induced platelet aggregation. Platelet-rich plasma (PRP) was incubated with 100–1000 *μ*M gallic acid or buffer for 3 minutes, and aggregation was induced by addition of (a) ADP (2.5 *μ*M) or (b) U46619 (1.5 *μ*M) (b). Gallic acid inhibited platelet aggregation induced by ADP or U46619 in a dose-dependent manner. These experiments are representative results of at least three similar experiments.

To test the influence of gallic acid on platelets, we performed *in vitro* platelet aggregation studies. PRP was incubated with different concentrations of gallic acid for 3 minutes before the addition of ADP or TxA_2_ analog U46619. Gallic acid significantly inhibited platelet aggregation induced by ADP (2.5 *μ*M) and U46619 (1.5 *μ*M) in a concentration-dependent manner (Figures 1(a) and 1(b)). The aggregation of platelets could not be fully inhibited by gallic acid. Gallic acid exerted no effects on the initial phase of platelet aggregation induced by ADP and U46619. The solvent control (0.5% DMSO) did not affect platelet aggregation stimulated by ADP or U46619 in either washed platelets or PRP (data not shown).

### 3.2. Gallic Acid Inhibits Platelet-Leukocyte Aggregates (PLAs)

**Figure 2:**
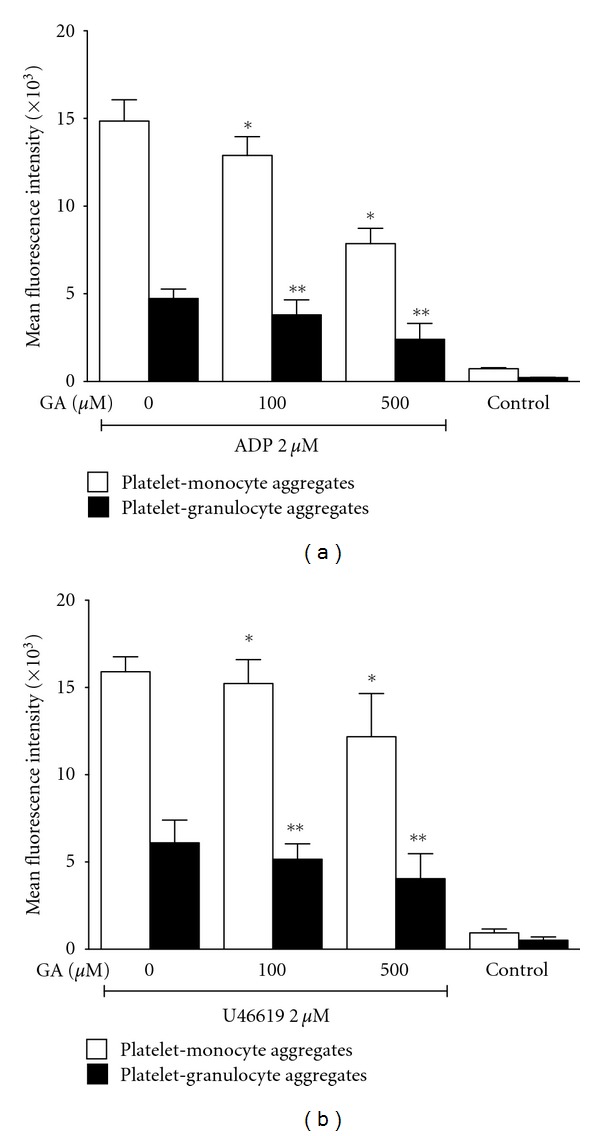
Flow cytometric analysis of the effects of gallic acid on platelet-leukocyte aggregates (PLAs) after stimulation with ADP or U46619. Quantitative results of the influence of gallic acid (100 *μ*M, 500 *μ*M) on PLA induced by (a) ADP (2 *μ*M) or (b) U46619 (2 *μ*M) by means of the mean fluorescence intensity of CD42a. Platelet-monocyte (open bars) and platelet-granulocyte (filled bars) aggregates were both inhibited by gallic acid in a concentration-dependent manner. Results are presented as mean ± SEM, *n* = 5. *,**P < 0.05 versus control samples stimulated with ADP or U46619. GA: gallic acid.

The influence of gallic acid on PLA was determined by flow cytometry in whole blood stimulated with ADP (Figure 2(a)) and U46619 (Figure 2(b)). The population of the granulocytes and monocytes was defined by size and the granularity. Monocytes were further probed by anti-CD14. The amount of platelets attached to the leukocytes was determined by the fluorescence of CD42a on granulocytes and monocytes. PLA increased significantly after adding ADP and U46619. Gallic acid (100 and 500 *μ*M) concentration-dependently inhibited ADP- and U46619-induced PLA in whole blood. 

### 3.3. Gallic Acid Inhibits P-Selectin Expression of Platelets

**Figure 3:**
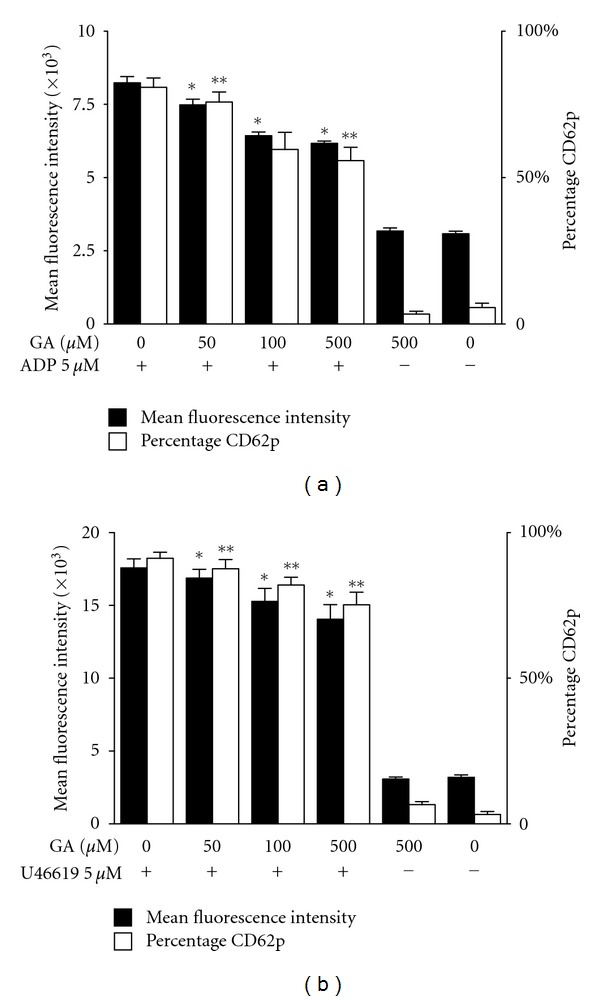
Inhibitory effects of gallic acid on CD62P expression of platelets stimulated with ADP or U46619. Platelets were pretreated with gallic acid (50 *μ*M, 100 *μ*M, and 500 *μ*M) and then stimulated with (a) ADP (5 *μ*M) or (b) U46619 (5 *μ*M). Surface expression of CD62P on platelets was quantified by flow cytometry. The percentage of platelets positive for CD62P expression (open bars) and the corresponding mean fluorescence intensity (MFI) (filled bars) of the positive platelets were determined. The results were presented as means ± SEM, *n* = 5. *,**P < 0.05 versus control samples stimulated by ADP or U46619. GA: gallic acid.

The influence of gallic acid on CD62P surface expression after stimulation of ADP (Figure 3(a)) and U46619 (Figure 3(b)) in PRP was measured by flow cytometry. The percentage of CD62P-positive platelets and MFI of CD62P on platelets was quantitatively assessed. Preincubation with increasing gallic acid concentrations (50, 100, and 500 *μ*M) had inhibitory effects of the P-selectin expression on platelets in response to ADP or U46619, and the inhibitory influence of gallic acid on platelets was concentration dependent.

### 3.4. Gallic Acid Inhibits Intracellular Ca^**2**+^ of Platelets

**Figure 4:**
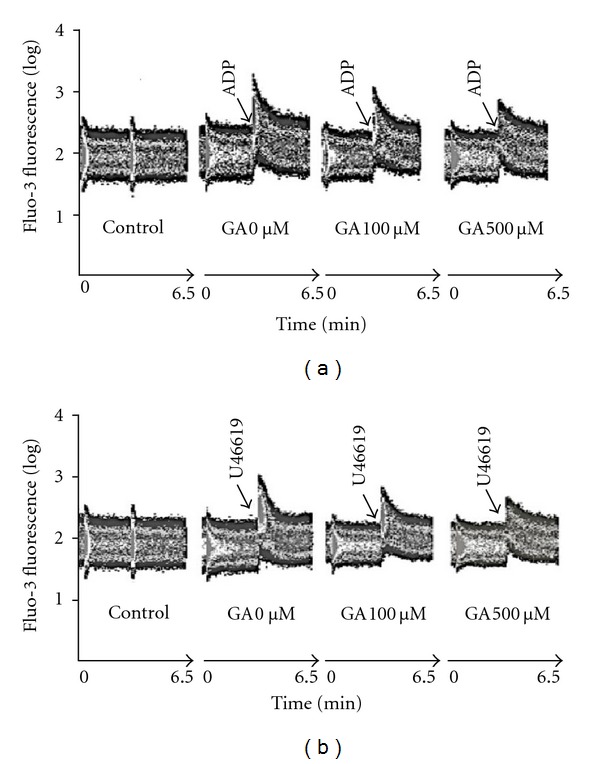
Effects of gallic acid on intracellular Ca^2+^ concentration of platelets measured by flow cytometry. Gallic acid at a concentration of 100 *μ*M and 500 *μ*M inhibited the intracellular Ca^2+^ rise, which were stimulated by (a) ADP (10 *μ*M) and (b) U46619 (2 *μ*M). These results were confirmed in 3 separate experiments. GA: gallic acid.

The effects of gallic acid on intracellular Ca^2+^ level of the platelets were studied by stimulation with ADP (Figure 4(a)) and U46619 (Figure 4(b)). As shown in Figure 4, ADP (10 *μ*M) and U46619 (2 *μ*M) could evoke a marked increase in Ca^2+^ concentration of platelets. Intracellular Ca^2+^ level of platelets was concentration-dependently inhibited by pre-incubation of gallic acid (100 *μ*M and 500 *μ*M). 

### 3.5. Effects of Gallic Acid on Activities of PKC*α*, P38MAPK, Akt, and GSK3*β*

**Figure 5:**
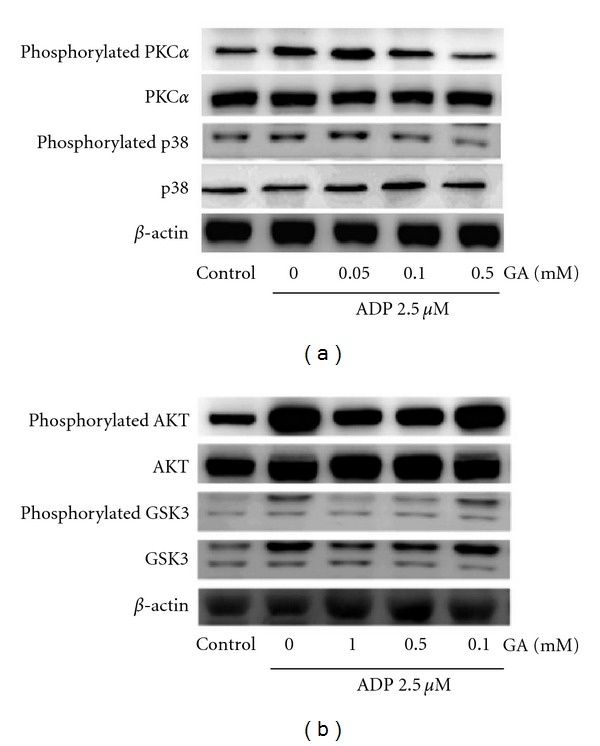
Effects of gallic acid on activation of protein kinase C alpha (PKC*α*), P38 mitogen-activated protein kinases (MAPK), Akt, and glycogen synthase kinase 3*β* (GSK3*β*) in platelets. Platelets were pretreated with gallic acid (50–1000 *μ*M) for 15 minutes prior to stimulation with ADP 2.5 *μ*M, and the phosphorylation of PKC*α* and p38 (a) and Akt and GSK3*β* (b) was assayed by western blot (*n* = 3). GA: gallic acid.

To evaluate the effects on the phosphorylation of P38 MAPK and PKC*α* from gallic acid, ADP (2.5 *μ*M) was added to PRP and the amount of the phosphorylated P38 MAPK and PKC*α* was assessed. The level of phosphorylated PKC*α* and p38MAPK of stimulated platelets increased apparently as compared with the other ones. Gallic acid (100 *μ*M, 500 *μ*M) could inhibit the phosphorylation of PKC*α* and p38MAPK in platelets receiving ADP stimulation (Figure 5(a)). In addition, the attenuation of PKC*α* phosphorylation from gallic acid manifested concentration-dependent manner. To assess the effects of gallic acid on Akt and GSK3*β*, ADP (2.5 *μ*M) was added to PRP and the level of phosphorylated Akt and GSK3*β* was determined (Figure 5(b)). The phosphorylation of Akt and GSK3*β* increased after stimulation of ADP as compared with other platelets, which was further reduced by gallic acid (1000 *μ*M, 500 *μ*M, and 100 *μ*M) concentration-dependently.

### 3.6. Effects of Gallic Acid on Reactive Oxygen Species of Platelets

**Figure 6:**
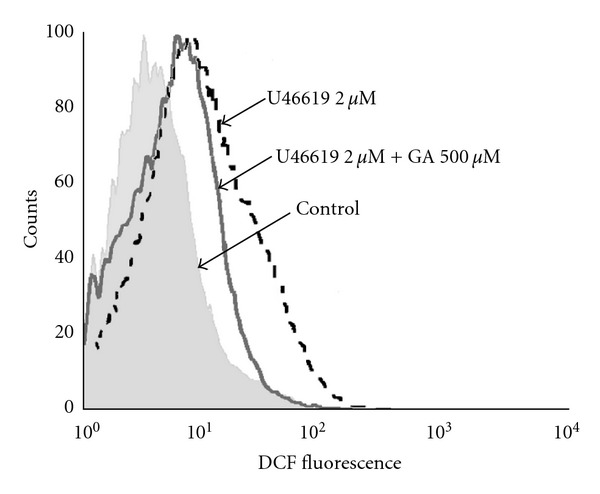
Effects of gallic acid on reactive oxygen species (ROS) production in platelets assessed by flow cytometry. Gallic acid at a concentration of 500 *μ*M had no inhibitory effects on platelet ROS stimulated with U46619 (2 *μ*M). This cytofluorimetric histogram of fluorescence of 2′7′-dichlorodihydrofluorescein (DCF) was representative of three similar experiments. GA: gallic acid.

To test whether the inhibitory effects of gallic acid on stimulated platelets came from antioxidant ability, the influence of ROS production after stimulation with U46619 and ADP was determined by the fluorescence of DCF. The representative histogram (Figure 6) showed that pretreatment of gallic acid (500 *μ*M) had no effects on DCF fluorescence compared to U46619 (2 *μ*M) treatment alone. There were no influences of ROS production after incubation of gallic acid followed by stimulation with ADP (data not shown). The result was indicative of gallic acid with no influence on ROS production of platelets stimulated with U46619 or ADP.

## 4. Discussion

Platelet aggregation and activation is a primary contributor to a variety of atherosclerotic diseases, including coronary artery disease, transplant vasculopathy, and carotid artery disease [3]. Antiplatelet therapies, including aspirin, cilostazol, and clopidogrel have been the mainstay of treatment for the atherosclerotic diseases. Gallic acid, a major constituent of red wine and tea, had been widely investigated for its cardiovascular protective properties. Our present study demonstrated for the first time that gallic acid could inhibit platelet aggregation, activation, and platelet-leukocyte aggregation and reduce Ca^2+^ mobilization, and this involved a decrease in the phosphorylation of PKC*α*/p38 MAPK and Akt/GSK3*β*. 

Platelet aggregation is known to be the result of the complex signal transduction cascades caused by the certain stimulants. Vessel wall injury triggers rolling and adhering of platelets to subendothelial matrix with their surface receptors. Subsequent platelet aggregation is the principle event in thrombus formation which plays a central role in the development of acute coronary syndrome [1, 2]. A semisynthetic antioxidant (hydroxy-tyrosyl gallate) related to gallic acid had been demonstrated to exert an inhibitory effect on platelet aggregation stimulated by thrombin [19]. Previous studies also reported that the antiaggregatory effects on platelets of red wine came from interference with the synthesis of TxA_2_, which served as an autocrine loop that accelerates aggregation [20]. Herein, we used *in vitro* models to show that gallic acid could reduce ADP- or U46619-stimulated aggregation of platelets in a concentration-dependent manner. Gallic acid could inhibit platelet aggregation stimulated by different proaggregatory stimulants, which had different action mechanisms responsible for platelet aggregation. This implied that gallic acid might block a common step shared by these agonists. 

PLA was found to increase in patients with acute coronary syndrome [21]. In various inflammatory clinical entities, such as cardiopulmonary bypass, hemodialysis, sepsis, and trauma, PLA level was higher than in the general population. PLA may trigger serial activation of platelets, which further leads to leukocyte recruitment into the vascular wall [22]. The formation of platelet-leukocyte conjugates is largely mediated by the binding of P-selectin expressed on activated platelets to PSGL-1 on leukocytes. Platelet P-selectin was also shown to play an indispensible role in arterial thrombogenesis by forming large stable platelet-leukocyte aggregates [22]. Recent reports demonstrate that gallic acid, as a structure-like molecule to P-selectin, can inhibit P-selectin-mediated adhesion both *in vitro* and in vivo [23]. Our study similarly showed that gallic acid could concentration-dependently reduce ADP- or U46619- induced PLA in whole blood. In our work, we further revealed that gallic acid could attenuate platelet P-selectin expression stimulated by ADP or U46619, which might further play a partial role in the inhibition of PLA formation. Collectively, gallic acid in our *in vitro* experiments was shown to regulate platelet aggregation, PLA formation, and platelet P-selectin expression induced by its stimulants, which might partially explain the cardiovascular protective effects of gallic acid.

Intracellular free Ca^2+^ concentration controls a number of platelet functions, including aggregation and P-selectin expression [24]. Platelet stimulants increase Ca^2+^ concentration, which consists of two components: release of Ca^2+^ from intracellular stores and Ca^2+^ entry through plasma membrane channels [24, 25]. Kim et al. found that pre-incubation of gallic acid with mast cells decreased the intracellular calcium level after provocation [26]. Hence, we further explored the influence of platelet Ca^2+^ concentration after incubation of gallic acid. Treatment of washed platelets with gallic acid of desired concentration (100 *μ*M and 500 *μ*M) could significantly reduce ADP- or U46619-evoked Ca^2+^ release. As Ca^2+^ is a potent stimulus of platelet granule secretion, the inhibition of Ca^2+^ release may lead to inhibition of platelet granule secretion and P-selectin expression [27]. This result did provide a mechanistic involvement by which gallic acid inhibited platelet aggregation and P-selectin expression. 

 Stimulation of platelets by proaggregatory agents results in phospholipase-C-(PLC-)-catalyzed hydrolysis of the plasma membrane phospholipid, phosphatidylinositol 4,5-bisphosphate, with concomitant formation of IP3 and diacylglycerol (DAG) [28, 29]. DAG can phosphorylate PKC, induce protein phosphorylation, ATP release, and intracellular Ca^2+^ rise, and finally activate platelets. In our work, ADP-induced PKC*α* phosphorylation was inhibited by gallic acid, suggesting that gallic-acid-mediated antiplatelet activity involved inhibition of PKC*α* activation. P38 MAPK provides a crucial signal that is necessary for aggregation of platelets caused by collagen or thrombin [30]. Among the numerous downstream targets of p38 MAPK, the most physiologically relevant one in platelets is cytosolic phospholipase A2 (cPLA2), which catalyzes arachidonic acid release to produce TxA_2_ [31]. Therefore, p38 MAPK appears to be necessary in TxA_2_-dependent pathways of platelet aggregation. Pre-treatment of gallic acid with the stimulated platelets reduced the phosphorylation of P38 MAPK in our study, which might also partially explain the inhibitory effects of gallic acid on platelet aggregation.

Platelet stimulated by agonists, for example, thrombin and ADP, could activate G protein-coupled receptors on platelet surface, which have been shown to activate multiple isoforms of PI3K and Akt [32, 33]. Platelets from Akt-1-deficient mice cannot form thrombus upon stimulation with thrombin and collagen [34]. In addition, glycogen synthase kinase (GSK) 3*β* (GSK3*β*) has been found in platelets and it can regulate platelet activation as an Akt effector [35, 36]. GSK3*β* is a ser-thr kinase that is regulated by its phosphorylation on ser9 [37]. Phosphorylation of this residue by Akt is related to decreased GSK3 activity, which releases a tonic inhibition of the GSK3 substrate. Therefore, the phosphorylation of GSK3*β* by Akt suppresses its inhibitory effect on platelet function. It was reported that decreased activity of GSK3*β* in haploinsufficiency mice or by treatment of platelets with GSK3*β* inhibitors (LiCl or SB216763) enhanced agonist-induced dense granule secretion [36]. Our results disclosed that gallic acid reduced the phosphorylation of Akt and GSK3*β* in platelets stimulated by ADP. Taken together, from our data, gallic acid may exert its antiplatelet effects via regulating the signals of PKC*α*/p38MAPK and Akt/GSK3*β*.

Both red wine and tea have been known to have antioxidant properties, but there were scarce studies about the anti-oxidant effects on platelet of gallic acid. Some studies even reported that gallic acid only had a weak inhibitory effect on oxidative stress [6]. Gallic acid was proved to have anti-oxidant effects on human lymphocytes and cardiac myocytes [12, 38]. In rat models, the intake of gallic acid was shown to be beneficial for the suppression of high fat diet-induced hepatosteatosis and oxidative stress [13]. Earlier investigations noticed that oxidative stress could activate platelets and lead to thrombosis through consumption of nitric oxide [39]. Reactive oxygen species also act as a secondary messengers that increase the cytosolic Ca^2+^ during the initial phase of platelet activation processes [40]. Contrarily, previous reports showed that gallic acid of similar concentrations could exhibit prooxidant effects [7, 41]. In our experiment, after pre-treatment of gallic acid, there was no influence on platelet ROS production with induction of U46619 (2 *μ*M) or ADP (2 *μ*M) (not shown). Therefore, the inhibitory results of platelet function from gallic acid might not come from the anti-oxidative actions. 

Though our present studies demonstrated that gallic acid had the possible cardiovascular protective roles from inhibiting platelet activation and its interaction with leukocytes, notably, gallic acid has been shown to induce apoptosis in tumor cells with higher sensitivity than that of normal cells in the comparative concentrations [7, 42, 43]. In fact, some of the concentrations of gallic acid used in our *in vitro* experiments are higher than those detected in plasma after acute intake of 50 mg gallic acid (as pure compound) or one cup of Assam black tea, which were within a low micromolar range (~4 *μ*mol/L) [5]. Nonetheless, gallic acid is largely consumed through tea, wine, nuts and even fruits in daily life [44, 45]. Studies are lacking that report the bioavailability after repeated intake of gallic acid. 

In conclusion, our study demonstrated that gallic acid inhibited platelet aggregation, P-selectin expression, and PLA formation stimulated by ADP or U46619, which is likely to be through decreasing intracellular Ca^2+^ mobilization. The inhibition of phosphorylation of PKC*α*/p38 MAPK and Akt/GSK3*β* in stimulated platelets after gallic acid pre-treatment was the suggestive mechanisms of action. This is the first report about the properties of gallic acid on platelet inhibition and its mechanisms. These findings of gallic acid suggest a possible therapeutic application of this agent in the diseases related to atherothrombosis.
